# Use of Fluorescence Spectroscopy and Chemometrics to Visualise Fluoroquinolones Photodegradation Major Trends: A Confirmation Study with Mass Spectrometry

**DOI:** 10.3390/molecules28020777

**Published:** 2023-01-12

**Authors:** Iván Sciscenko, Paula García-Negueroles, Ana María Amat, Isabel Oller, Carlos Escudero-Oñate, Laura Ferrando-Climent, Antonio Arques

**Affiliations:** 1 Departamento de Ingeniería Textil y Papelera, Universitat Politècnica de València (UPV), Plaza Ferrándiz y Carbonell s/n, 03801 Alcoy, Spain; 2 CIEMAT-Plataforma Solar de Almería, Carretera de Senés km 4, 04200 Tabernas, Spain; 3 Institute for Energy Technology (IFE), Instituttveien 18, 2007 Kjeller, Norway

**Keywords:** antibiotics, low-cost analysis, pharmaceuticals, photolysis, seawater, transformation products, water treatment

## Abstract

In this work, we employed EEM-PARAFAC (fluorescence excitation-emission matrices-parallel factor analysis) as a low-cost tool to study the oxidation pathways of (fluoro)quinolones. Amounts of 12.5 μM of enrofloxacin (ENR), ciprofloxacin (CIP), ofloxacin (OFL), oxolinic acid (OA), and flumequine (FLU), as individual solutions, were irradiated under UVA light. A 5-component PARAFAC model was obtained, four of them related to the parent pollutants, named as ENR-like (including CIP), OFL-like, OA-like, and FLU-like, and an additional one related to photoproducts, called ENRox-like (with an emission red-shift with respect to the ENR-like component). Mass spectrometry was employed to correlate the five PARAFAC components with their plausible molecular structures. Results indicated that photoproducts presenting: (i) hydroxylation or alkyl cleavages exhibited fingerprints analogous to those of the parent pollutants; (ii) defluorination and hydroxylation emitted within the ENRox-like region; (iii) the aforementioned changes plus piperazine ring cleavage emitted within the OA-like region. Afterwards, the five antibiotics were mixed in a single solution (each at a concentration of 0.25 μM) in seawater, PARAFAC being also able to deconvolute the fingerprint of humic-like substances. This approach could be a potential game changer in the analysis of (fluorescent) contaminants of emerging concern removals in complex matrices, giving rapid visual insights into the degradation pathways.

## 1. Introduction

Quinolones and fluoroquinolones ((F)Qs) are synthetic broad-spectrum antibiotics widely used in animal farms, aquaculture facilities, and human medicine, which, together with cephalosporins and macrolides, are catalogued as “highest priority” medicines by the World Health Organization [1]. These antibiotics reach the environment in many different ways: (i) manure disposal, (ii) direct discharge (e.g., through fish farms), and (iii) wastewater treatment plants effluents and activated sludge, used as fertilizer [2,3]. In fact, (F)Q behaviour within wastewater treatment plants is considered a major concern for the European Union; according to the Joint Research Centre 2019 report, it is estimated that ciprofloxacin environmental loadings’ reduction will be negligible even after new water directive enforcement (85% of the ciprofloxacin load incoming to a wastewater treatment plant is retained in the sludge) [4]. Therefore, it is not surprising to see that (F)Qs are ubiquitous in surface and groundwater worldwide, such as the Ter river (Spain), as reported by Ferrando-Climent et al. [5], finding the highest global median values in China and India natural waters [6].

Due to their presence in surface waters, the photolysis of these compounds has been thoroughly studied. For instance, it was reported that they might suffer slight molecular changes under light irradiation, where they could even transform into another commercial (F)Q, as is the case of the aforementioned ciprofloxacin (used in human medicine), the main photoproduct of enrofloxacin (generally employed for veterinary uses) [7]. (F)Q photolytic rate constants and degradation pathways are highly pH and cation dependent. They exhibit the greatest photolysis rates at neutral pH, when the neutral form (and zwitterionic when also presenting amino groups) is the most abundant [8,9], whereas regarding the cations, it is variable, depending on the type of cation and (F)Q, respectively, forming diverse (F)Q–metal complexes with different stabilities, observing photolytic enhancement [10] or hindrance [11].

When analysing the photolysis of (F)Qs (or degradation through an advanced oxidation process), several transformation products are formed, being necessary to monitor not only the removal of the parent compounds, but also the respective kinetics of the formed by-products. However, the existing analytical methodologies suitable for monitoring the whole process (parent compound and transformation products) are based on expensive technologies, such as ultra-high performance liquid chromatography coupled with high resolution mass spectrometry (UPLC-HRMS), which also requires highly skilled personnel to deliver the analytics.

In recent studies carried out by the authors [12,13,14], EEM-PARAFAC (fluorescence excitation emission matrices-parallel factor analysis) proved to be an economic, reagent-free (although buffers might be used), and easy to handle tool to study oxidations of (F)Qs. PARAFAC is a chemometric tool, usually defined as a more simple and restricted version of principal component analysis (it uses fewer degrees of freedom than the latter) [15], mostly employed to deconvolute overlapping fluorescent fingerprints from the different fluorophores contained within a EEM [16]. This property allows the user to measure the individual signal intensities of the respective fluorophores contained in a sample without a separative method (e.g., HPLC). This advantage was therefore employed to study the degradation process of single [12] and mixtures of (F)Qs [13,14], results showing that it was possible to track the fluorescence intensity of the parent pollutants and hypothesize on the structure of formed by-products, based on the fluorescence fingerprints changes and their intensity evolution during the oxidation process. However, in the former studies, EEM-PARAFAC was never qualified against UPLC-HRMS, where the latter can support the hypothesis made from a PARAFAC model.

**Figure 1:**
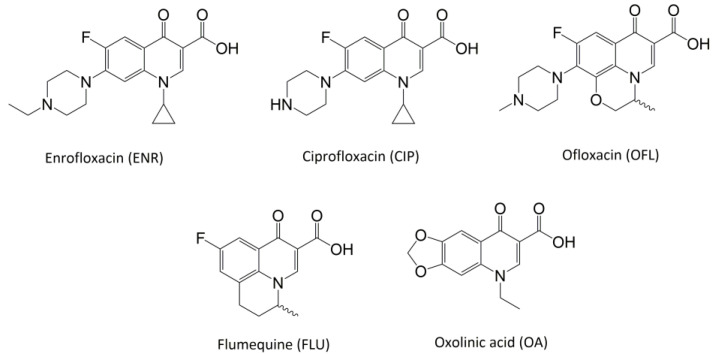
Studied (fluoro)quinolones in this work.

In line with the above mentioned statements, in this study, we employed EEM-PARAFAC to analyse the photolytic degradation of five (F)Qs, as individual and mixed solutions, of enrofloxacin (ENR), ciprofloxacin (CIP), ofloxacin (OFL), flumequine (FLU), and oxolinic acid (OA) (see Figure 1). We aimed to correlate the fluorescence time-evolution of the different PARAFAC-components (fluorophores) found at the corresponding analysis with the molecular structures of identified photoproducts, with their respective formation kinetics, using UPLC-HRMS (Orbitrap-based technology). Therefore, our work intended to verify the possibility of predicting the photodegradation pathways of (F)Qs by employing EEM-PARAFAC instead of expensive and sophisticated equipment. This approach could be a potential game changer in the analysis of fluorescent compound degradation, as well as in other applications such as stability assessments of pharmaceuticals [17] or beverages [18], allowing the simultaneous measurement of fluorescence intensity trends from several parent compounds and their formed by-products in complex matrices, rapidly visualising the overall behaviour of the fluorescent organic matter from a specific system.

## 2. Results and Discussion

### 2.1. Photolysis of Individual Solutions

Firstly, individual solutions of ENR, CIP, OFL, OA, and FLU, each at a concentration of 12.5 µM, were irradiated with UVA light. Although the chosen concentration was 2–3 orders of magnitude higher than the one normally found in urban wastewater and natural effluents [3], the purpose was to create a high signal-to-noise ratio from the formed by-products in order to obtain an accurate structure elucidation by UHPLC-HRMS. The water matrix consisted of ultra-pure water at pH = 7.5 (adjusted with NaOH 10 mM).

To study the degradations pathways, three analytical methods were employed: (i) parent pollutants removals were measured with HPLC coupled to UV/vis; (ii) the degradation trends of families of compounds (those presenting comparable molecular structures and exhibiting the same fluorescence fingerprint) were followed with EEM-PARAFAC; and (iii) by-product molecular structures were tentatively elucidated with mass spectrometry analysis.

#### 2.1.1. HPLC-UV/Vis Measurements

**Figure 2:**
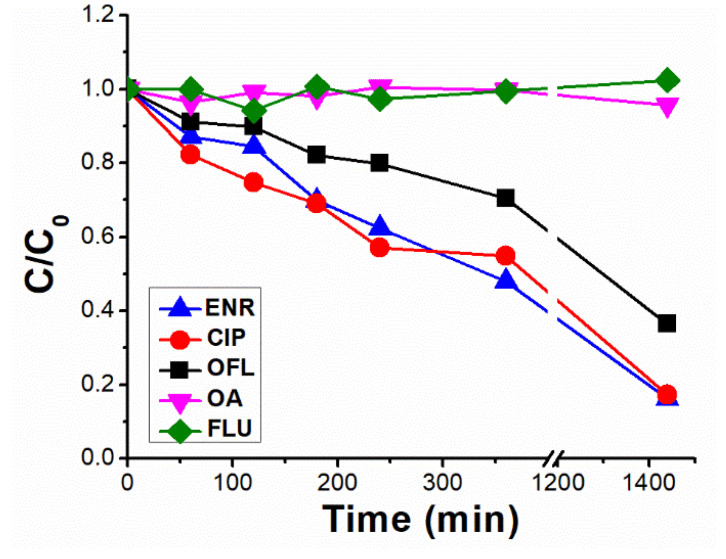
Normalised photolytic degradation under UVA irradiation of enrofloxacin (ENR), ciprofloxacin (CIP), ofloxacin (OFL), oxolinic acid (OA) and flumequine (FLU) individual solutions, each with an initial concentration of 12.5 µM, spiked in ultra-pure water at pH = 7.5.

As shown in Figure 2, photolysis trends (ENR = CIP > OFL > OA = FLU) are in line with the different photo-stabilities of (F)Qs reported elsewhere [8,19,20,21]: OA and FLU being the most stable ones, both exhibiting negligible photodegradation percentages after 24 h of irradiation (due to lack of piperazine ring), followed by OFL with 64% removal in 24 h (presence of piperazine ring, but with an oxygen group bonded to the aromatic ring), and ENR and CIP, both with 85% in 24 h (with piperazine ring and without electron donor groups bonded to the aromatic ring, being more photolabile).

#### 2.1.2. Fluorescence Spectroscopy Measurements

**Figure 3:**
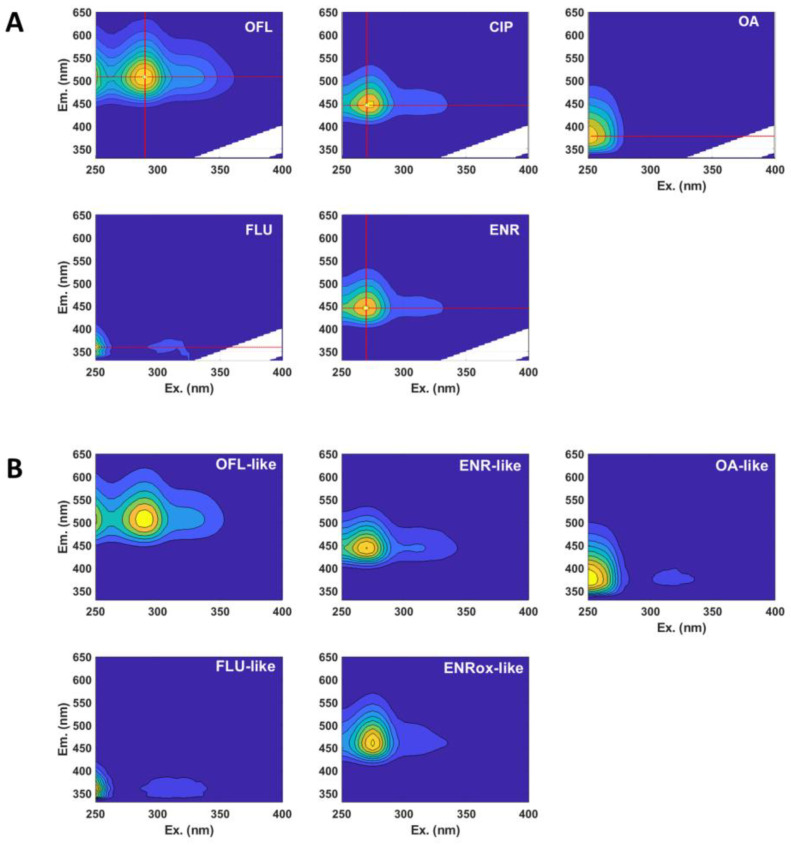
(**A**) Normalised fluorescence excitation-emission matrices (EEM) of (F)Qs standard solutions; (**B**) obtained PARAFAC model. The component ENR-like includes CIP fluorescence (due to its analogous fingerprint with ENR), and ENRox-like represents the fluorescence of a family of major transformation products coming from ENR and CIP photolysis.

After measuring the EEM from the different photolysis time-intervals of ENR, CIP, OFL, OA, and FLU (whose EEM prior to irradiation, are shown in Figure 3A), a PARAFAC model of five components was obtained (see Figure 3B). 

Enrofloxacin and ciprofloxacin fluorescence were described by the same component (called ENR-like, with λ_ex1_ = 270 nm, λ_ex2_ = 310 nm, and λ_em_ = 440 nm) as they have identical fingerprints (Figure 3A). On the other hand, the analogous of ofloxacin, oxolinic acid and flumequine were easily deconvoluted by the algorithm, and accordingly called OFL-like (λ_ex1_ = 290 nm, λ_ex2_ = 325 nm, and λ_em_ = 505 nm), OA-like (λ_ex1_ < 250 nm, λ_ex2_ = 315 nm, and λ_em_ = 372.5 nm), and FLU-like (λ_ex1_ < 250 nm, λ_ex2_ = 315 nm, and λ_em_ = 360 nm). The additional component, named ENRox-like (λ_ex1_ = 275 nm, λ_ex2_ = 340 nm, and λ_em_ = 457.5 nm), was found from generated photoproducts, whose fingerprint exhibited a fluorescence maximum at slightly higher wavelengths than ENR-like (red shift). Therefore, there is a family of by-products (not present in the initial samples) emitting within the region of ENRox-like.

The latter component was also found in a previous work, where a mixture consisting of enrofloxacin, ofloxacin and sarafloxacin (another commercial fluoroquinolone, whose fluorescent fingerprint is comparable to ENRox-like) was photolysed under simulated sunlight at neutral pH in ultra-pure water, detecting sarafloxacin-like component scores (i.e., fluorescence intensity) increment, indicating that the formed by-products from the three parent pollutants were emitting in this region [13]. When employing more oxidative conditions, e.g., photo-Fenton [22], a comparable component to ENRox-like was also found, but exhibiting a global maximum shift of 15 nm towards shorter wavelengths (blue shift) instead [12,14].

**Figure 4:**
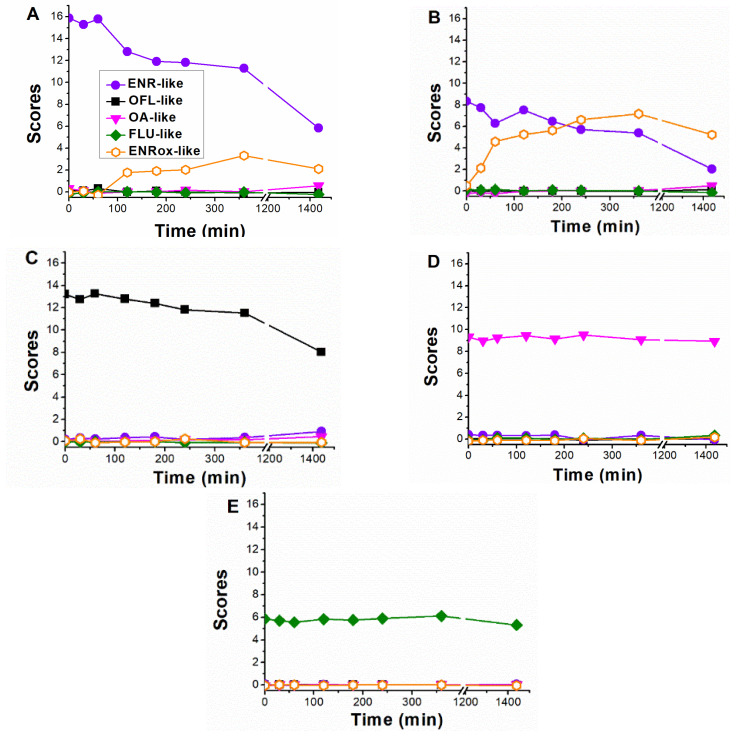
PARAFAC components evolution obtained for the different photolysis experiments: (**A**) enrofloxacin (ENR), (**B**) ciprofloxacin (CIP), (**C**) ofloxacin (OFL), (**D**) oxolinic acid (OA), and (**E**) flumequine (FLU). The initial score values for ENR-like, OFL-like, OA-like, and FLU-like reflect the fluorescence intensity differences from the studied (F)Qs.

When analysing the scores time-evolution of the five PARAFAC components during ENR and CIP photolysis, comparable trends were observed in both cases (Figure 4A and B, respectively), being the initial ENR-like scores of CIP lower than those of ENR, in line with the lower fluorescence emission of the first one [14,23].

Results from enrofloxacin photolysis show that the ENR-like scores exhibited a significantly lower decay than the respective pollutant removal rate (61% in 24 h of ENR-like scores reduction, previously observing an 85% for ENR concentration decay in Figure 2), related to the fact that ENR-like also considers the fluorescence from those photoproducts with analogous fingerprints to the parent compound. On the contrary, in the case of CIP photolysis, ENR-like scores decay did not exhibit a significative difference with the respective CIP chromatographic area decay (compare Figure 4B with Figure 2). We can therefore conclude that, during CIP photolysis, the generated by-products do not emit within the ENR-like region. In line with this observation, the ENRox-like scores increment rate was considerably higher in the case of CIP photolysis (reaching a score value of 7.2 in 360 min) than in the case of ENR (reaching a score value of 3.3 in 360 min).

Since CIP is reported as a major photoproduct of ENR [7,24], also confirmed in this work and discussed in Section 2.1.3, the formation of further oxidized by-products is expected to be higher if the starting compound is CIP, rather than ENR. In addition, there was a slight increment of OA-like scores in both cases, only significant after 24 h of irradiation (OA-like score value of 0.5 in 24 h) indicating formation of highly oxidized photoproducts.

For the OFL photolysis (Figure 4C), the EEM changes observed indicate that the OFL-like component scores decay was 40% in 24 h (slower than the OFL individual degradation rate, 64% in 24 h, as shown in the previous section), and a slight increase in ENR-like and OA-like. 

As expected, OA-like and FLU-like fluorescence trends did not show significative changes with respect to OA and FLU (see Figure 4D,E), as their respective photolysis scores were negligible.

#### 2.1.3. Mass Spectrometry Measurements: Correlating EEM-PARAFAC Components with MS Tentative Molecular Structures

**Figure 5:**
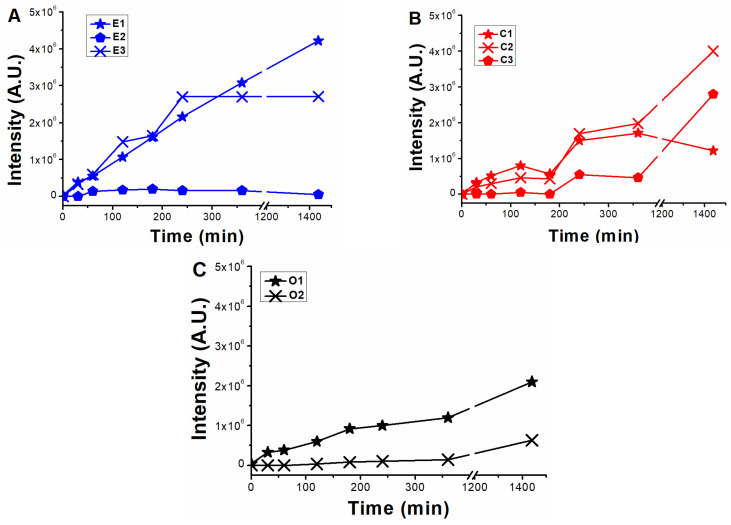
Photoproduct formation during photolysis of 12.5 µM standard solutions of: (**A**) enrofloxacin (formation kinetics of E1–E3), (**B**) ciprofloxacin (formation kinetics of C1–C3), and (**C**) ofloxacin (formation kinetics of O1 and O2). The signal intensity for the photoproducts found from oxolinic acid and flumequine photolysis were negligible in both cases, and results are not shown.

Several transformation products were identified from ENR, CIP, and OFL photodegradation experiments (Table 1). The identified photoproducts showed defluorination, alkyl cleavages, hydroxylation, and piperazine ring cleavage, typical of (F)Qs [21,25,26], and followed a kinetic pattern for their generation, shown in Figure 5. For OA and FLU, only a single photoproduct for each compound was detected (named Oa1 and F1, respectively), both with scarce intensity within their respective chromatograms, corroborating the low degree of photolysis previously observed.

During the photolysis of ENR, three major photoproducts were generated: E1 (ethyl cleavage from piperazine ring, producing CIP molecules), E2 (internal cleavage of the bond between one nitrogen and carbon of the piperazine ring), and E3 (defluorination and subsequent double hydroxylation). Comparable to E3, photoproduct C1, found during CIP photolysis, exhibits analogous double hydroxylation and fluoride elimination. The C2 intermediate can be analysed as a more oxidized version of E2; C3 is a compound with a considerable degree of oxidation (substitution of piperazine ring by a hydroxy group and rupture of cyclopropyl moiety). Regarding the new identified photoproducts, E3 and C3, although their exact molecular structure was not found in other works, their structures are in agreement with the ones shown in the literature. For E3, double hydroxylation of the quinolonic core with fluoride substitution during ciprofloxacin photolysis was observed in a similar way [27], whereas for C3, substitution of the piperazine ring and fluoride by hydroxyl groups was also observed during enrofloxacin photolysis [28], but without the cyclopropyl ring rupture.

Figure 4 was compared with Figure 5 to evaluate the molecular structures of the PARAFAC components. As mentioned in the previous section, the slower fluorescence decay observed with EEM-PARAFAC compared to HPLC-UV/vis during ENR and OFL photolysis measurements is attributed to the contribution of slightly oxidized by-products exhibiting analogous fluorescence fingerprints to the parent pollutant, which is the case of E1 (CIP) and possibly E2 in enrofloxacin photolysis, as well as for O1 and O2 in ofloxacin photolysis, emitting in the regions of ENR-like and OFL-like, respectively.

When analysing the scores of ENRox-like, the plausible structures contributing to the fluorescence increment of this component might be E3, C1 and/or C2. On one hand, as was previously stated, since ENR-like scores exhibit trends analogous to CIP degradation (see Figure 2 and Figure 4B), it can be assumed that the photoproducts formed during CIP photolysis, C1 and C2, should not contribute significantly to the ENR-like fluorescence region. Moreover, since E3 and C1 present comparable structures to one another, E3 should also emit in the region of the ENRox-like component. In line with these statements, intensity evolution from mass spectrometry analysis of E3 (Figure 5A) and C2 (Figure 5B) exhibited comparable trends to ENRox-like during ENR and CIP photolysis (Figure 4A,B, respectively). Therefore, defluorination and/or hydroxylation of (F)Qs could produce maximum emission shifts towards higher wavelengths.

On the other hand, to explain the scores increment from OA-like component during ENR and CIP photolysis (only observable after 24 h of irradiation), the photoproducts emitting within this region should be similar to C3, which is a molecule highly similar to the one of oxolinic acid (see Figure 1), with double hydroxylation in the aromatic ring, rupture of cyclopropyl moiety, and without the fluoride and piperazine ring moieties from fluoroquinolones. This is also in agreement with two previous works: (i) when following the degradation of ENR alone by photo-Fenton, a PARAFAC component with emission maximum at λ_ex_ < 250 nm and λ_em_ ≈ 380 nm was observed [12], and (ii) when degrading a mixture of ENR, OFL, CIP, FLU, and OA with solar-photo-Fenton, there was observed a significant OA-like scores increment, and it was assumed that the formed oxidation by-products had molecular structures similar to OA [14].

### 2.2. Photolysis of (F)Q Mixture

Closer to realistic conditions, another set of photolysis was carried out, with solutions containing the mixture of the five (F)Qs, each in a concentration of 0.25 μM ([(F)Q total] = 1.25 µM). Seawater was chosen as a natural environment where (F)Qs are frequently present due to its extended use in aquaculture facilities [35,36].

**Figure 6:**
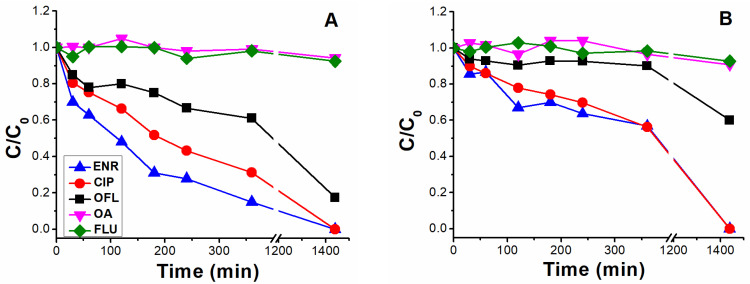
Photolysis of solutions containing the five (fluoro)quinolones ((F)Qs) mixed, each one with an initial concentration of 0.25 µM, into (**A**) ultra-pure water and (**B**) seawater.

In general, observed photolytic rates were slower in seawater than in ultra-pure water, related to the lower transmittance of the former as well as to the presence of other water constituents, such as carbonates and bicarbonates, reactive oxygen species (self-sensitized from (F)Qs and dissolved organic matter [9,37]) scavengers. For instance, at 360 min in ultra-pure water, 65% degradation was observed for ENR (Figure 6A), compared to 40% in the same period in seawater (Figure 6B). For OFL, the respective removal percentages at 360 min were 30% and 10%, respectively, and for OA and FLU percentages were <5% in both water matrices. Interestingly, since CIP is a major photoproduct of ENR, the observed slower CIP removal in ultra-pure water could be explained by its parallel formation from ENR (differently from when it was carried out as individual solution, were ENR and CIP exhibited comparable photolysis rates (see Figure 2)). However, in seawater, comparable kinetics between ENR and CIP were observed, most likely linked to their slower removal rates.

To simultaneously analyse the degradation of all pollutant and by-product evolution, a EEM-PARAFAC model was carried out, obtaining the five analogous components shown in Figure 3B, but now including an additional one belonging to humic-like substances present in the seawater, with maximum located at λ_ex_ < 250 nm and λ_em_ = 475 nm [14,38].

**Figure 7:**
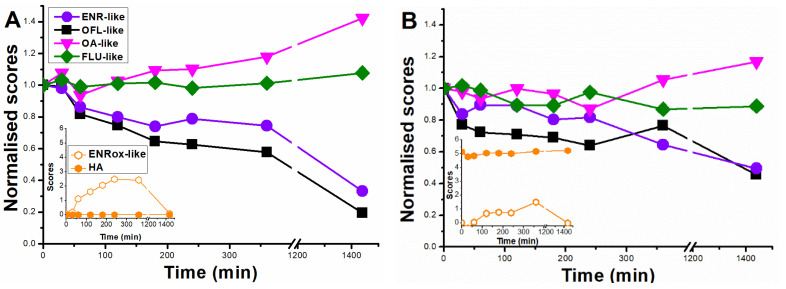
PARAFAC trends of the six deconvoluted fluorescent fingerprints during the photolysis of (fluoro)quinolones mixtures (0.25 µM of enrofloxacin, ciprofloxacin, ofloxacin, oxolinic acid, and flumequine, respectively) in (**A**) ultra-pure water and (**B**) seawater. The PARAFAC components described the fluorescence kinetics of the following families of compounds: ENR-like (fluorescence from enrofloxacin, ciprofloxacin/E1, and E2 compounds); OFL-like (OFL, O1, and O2); OA-like (OA, Oa1, and C3); FLU-like (FLU and F1); ENRox-like (E3, C1, and C2); and HA (humic-like substances).

Results in ultra-pure water (Figure 7A) showed that: (i) ENR-like (ENR, CIP/E1, and E2 compounds) exhibited a decay of 70% in 24 h; (ii) OFL-like (kinetics of OFL plus photoproducts O1 and O2) score decay was 80% in 24 h; (iii) OA-like (kinetics of OA, Oa1, and C3 compounds) scores increased by 40% in 24 h; (iv) FLU-like (kinetics of FLU and F1 compounds) scores remained practically constant the whole experiment; (v) ENRox-like (kinetics of by-products E3, C1, and C2) scores exhibited maximum formation at 360 min, with a subsequent decay, being negligible after 24 h of irradiation. In line with the absence of humic-like substances (named as HA), their scores remained as zero.

Looking into the results obtained in seawater (Figure 7B), the overall scores followed analogous trends to the ones obtained in ultra-pure water, but with slower rates, in agreement with concentration kinetics (Figure 6). Fluorescence from HA was constant for 24 h, indicating that the UVA light was not energetic enough to degrade it.

## 3. Materials and Methods

### 3.1. Reagents

High purity (>99%) FLU, OA, OFL, ENR, and CIP were purchased from Sigma-Aldrich. NaOH, H_2_SO_4_ (96%), glacial acetic acid, and UHPLC-grade methanol and acetonitrile were provided by AppliChem-Panreac. Ammonium acetate was provided by Scharlau.

Respective (F)Q 250 µM stock solutions were prepared in basic media; all solutions were stable at dark conditions (no hydrolysis was observed).

### 3.2. Irradiations

Two sets of experiments were carried out, one consisting of irradiating the individual standard solutions of ENR, CIP, OFL, OA, and FLU, with an initial concentration of 12.5 μM in ultra-pure water at pH 7.5, and another one containing the former compounds mixed in a single solution (each in concentration 0.25 μM) of ultra-pure water at initial pH 7.5, and a single solution of seawater (taken from Oslo’s fjord, Norway, and with the following characteristics: pH = 7.6, conductivity 1400 µS/cm, total inorganic carbon = 4.5 mg/L, total organic carbon = 6.0 mg/L, absorbance 365 nm = 0.036).

Irradiations were always performed for 24 h with an ultraviolet-A (UVA) bulb lamp ONASC (1.5 W, 230 V, and 365 nm emission intensity maximum), taking intermediate samples at different time intervals. The lamp was placed 3 cm above the testing solution surface and centred inside the reactor (2 L total volume, filled with 1.5 L of solution). Aluminium paper surrounded the reactor to collect incident radiation and distribute the reflected light homogeneously.

Samples were taken at different time intervals, always filtered by 0.45 μm PTFE filters (Chromafil Xtra). As performed in previous works [13,14], samples analysed by fluorescence spectroscopy were previously buffered at pH = 4.0 (employing an acetic acid–acetate solution), to avoid fluorescence fluctuations due to pH changes, this also being a pH value where these compounds exhibit maximum fluorescence quantum yields [8]. Samples from the solutions containing single (F)Q compounds 12.5 μM irradiations were required to be diluted with a factor 1:5, whereas samples from the mixture degradation were not.

### 3.3. Chemical Analysis

(F)Q photolysis rates were measured employing an UHPLC Agilent 1290 Infinity II (Rocklin, CA, USA), coupled with fluorescence and UV/vis detectors, and employing a reverse phase column (Waters Acquity, 50 mm × 2.1 mm, 1.7 µm). A gradient elution mode (flow rate 0.5 mL/min) was employed as following: formic acid 0.1%, methanol and acetonitrile, initial 85:7.5:7.5, respectively (in % *v*/*v*), for the first 6 min, afterwards increasing non-polar fraction in 4 min to 7.5:7.5:85, remaining constant for an additional minute, and changing the solvent fractions back to the initial values during the next 2 min. When measuring removals from the (F)Q standard solutions (initial concentration 12.5 µM), the UVA/vis detector was employed, using 285 nm fixed wavelength for ENR, CIP, and OFL determinations, and 250 nm for OA and FLU. When degrading the (F)Q mixture solutions (solutions containing the five (F)Q together, each one with initial concentration 0.25 µM), the fluorescence detector was employed instead, employing λ_ex_ = 285 nm/λ_em_ = 480 nm for ENR, CIP, and OFL determinations, and λ_ex_ = 312 nm/λ_em_ = 366 nm for OA and FLU.

EEM determinations were carried out with a Horiba PTI Quanta Master 400 (Kyoto, Japan) spectrofluorometer, employing an excitation range of 250–400 nm (recorded with 5 nm intervals) and emission range of 330–650 nm (recorded within 2.5 nm intervals).

Mass spectrometry analysis was carried out with UPLC-HRMS (QExactiveTM- Thermo Fisher Scientific, MA, USA), employing a Sherzo-Imtakt C18 column (150 mm × 20 mm, 5 μm). Mobile phase formic acid 0.1%: acetonitrile was used in gradient elution from 90:10 to 10:90 in 15 min. The entire system was controlled via Xcalibur 3.0 software (Thermo Fisher Scientific, Waltham, MA, USA). Analytical parameters were as follows: 50–700 *m*/*z* range; ionization voltage, 3.5 kV; heater temperature, 300 °C; capillary temperature, 350 °C; sheath gas flow, 40 arb; auxiliary gas flow, 20 arb; collision energy, 55 eV in higher-energy collisional dissociation; dynamic exclusion, 10 s; and isolation window, 2 Da. Heated electrospray ionization was employed in positive mode only (no appreciable signals were observed when ionizing with negative mode). Samples were recorded in full-scan mode within a mass-to-charge (*m*/*z*) range of 50 to 700 *m*/*z* at a resolving power of 70,000 FWHM (MS). Ion fragmentation was performed in data-dependent acquisition (DDA) mode for the three most intense ions (TOP 3) at a resolving power of 35,000 FWHM (MS/MS). Computational data files were processed through Thermo Scientific Q-Exactive 2.0 (Thermo Fisher Scientific, Waltham, MA, USA). The instrument was always calibrated to previous measurements, employing Pierce LTQ Velos ESI Positive Ion Calibration Solution (Thermo Fisher Scientific, Waltham, MA, USA) and Pierce Negative Ion Calibration Solution (Thermo Fisher Scientific, Waltham, MA, USA) for positive and negative modes, respectively.

Absorbance spectrums were measured with a Hitachi-UH5300 (Tokyo, Japan) spectrophotometer, and seawater total organic and inorganic carbon measurements were carried out with a Shimadzu TOC-V (Kyoto, Japan).

### 3.4. EEM-PARAFAC Modelling

Two PARAFAC models were performed, one with the dataset containing the EEM from the photolysis of standard solutions of 12.5 μM of ENR, CIP, OFL, FLU, and OA, respectively, and the other employing the dataset containing the former EEM plus the ones taken during photolysis of (F)Q mixtures in ultra-pure water and seawater; the first dataset consisted of 40 EEM and the second of 56. Blanks were subtracted by uploading the ultra-pure water EEM, signal intensity normalization was corrected with water Raman scatter signal intensity at 350 nm, and the inner filter effect was corrected with respective absorbance spectrums. PARAFAC analysis was performed employing MATLAB 2021a software with the free and user friendly graphical user interface, EEMlab [39], employing the drEEM toolbox [16]. Data pre-processing and modelling was performed as described in previous works [12,40].

## 4. Conclusions

EEM-PARAFAC was successfully employed to track, simultaneously, the fluorescence from parent pollutants and formed by-products during irradiation with UVA light of ENR, CIP, OFL, OA, and FLU, individually and as a mixture. High resolution mass spectrometry results were used to assign the molecular structures of formed photoproducts to the respective PARAFAC components, allowing EEM-PARAFAC to predict the types of by-products being formed.

The main photoproducts exhibited defluorination, cleavage of alkyl (methyl and ethyl) groups, piperazine ring oxidation and cleavage, and hydroxylation, coming from ENR, CIP, and OFL photolysis, since the photolysis of OA and FLU was negligible. When the degradation degree was low, emissions remained in the EEM region of the respective parent pollutants, while when the oxidation was considerable (defluorination, piperazine ring cleavage and more than one hydroxylation), the fluorescence maximums of ENR, CIP, and OFL shifted to shorter wavelengths, as was the case of photoproduct C3 (with a molecular structure similar to OA), explaining the observed OA-like scores increment during the photolysis of the aforementioned (F)Qs.

This study confirms the hypothesis and assumptions previously made in our related works. This is an important result, as it shows that, with a low-cost equipment, degradation pathways of compounds in low concentrations can be rapidly elucidated; this is also applicable to any changing system involving fluorescent molecules.

## Figures and Tables

**Table 1:** EEM-PARAFAC correlation with UHPLC-HRMS results. The transformation products were named with a first letter associated to the compound which were generated, and a number linked to the oxidation degree: E1–E3, from Enrofloxacin; C1–C3, from Ciprofloxacin; O1 and O2, from Ofloxacin; F1, from Flumequine; and Oa1, from Oxolinic acid.

Photoproduct Name	Experimental Mass from [M-H]^+^ (g/mol)	Molecular Formula	Tentative Molecular Structure	Reported by	Associated PARAFAC Component
E1	332.14050	C_17_H_18_O_3_N_3_F	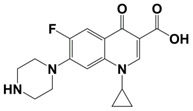	[7,24,28]	ENR-like
E2	334.15615	C_17_H_20_O_3_N_3_F	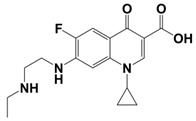	[28,29]	ENR-like
E3	374.17105	C_19_H_23_O_5_N_3_	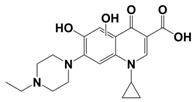	---	ENRox-like
C1	346.14114	C_17_H_19_O_5_N_3_	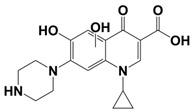	[27,30]	ENRox-like
C2	316.13034	C_16_H_17_O_4_N_3_	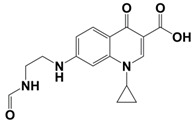	[30,31]	ENRox-like
C3	250.07191	C_12_H_11_O_5_N	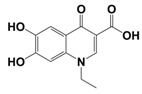	---	OA-like
O1	348.13541	C_17_H_18_O_4_N_3_F	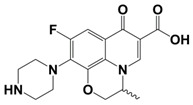	[9,32]	OFL-like
O2	364.13229	C_17_H_18_O_5_N_3_F	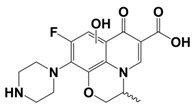	[32]	OFL-like
F1	276.06589	C_14_H_10_O_4_NF	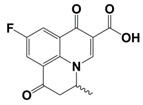	[33]	---
Oa1	278.07141	C_13_H_11_O_6_N	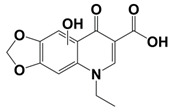	[34]	---

## Data Availability

Not applicable.
